# Prediction of delirium occurrence using machine learning in acute stroke patients in intensive care unit

**DOI:** 10.3389/fnins.2024.1425562

**Published:** 2025-01-09

**Authors:** Hyungjun Kim, Min Kim, Da Young Kim, Dong Gi Seo, Ji Man Hong, Dukyong Yoon

**Affiliations:** ^1^Department of Biomedical Informatics, Ajou University School of Medicine, Suwon, Republic of Korea; ^2^MDHi Corp, Suwon, Republic of Korea; ^3^Department of Neurology, Ajou University School of Medicine, Suwon, Republic of Korea; ^4^Department of Convergence Healthcare Medicine, Graduate School of Ajou University (ALCHeMIST), Suwon, Republic of Korea; ^5^Department of Biomedical Science, Ajou University Graduate School of Medicine, Suwon, Republic of Korea; ^6^Department of Biomedical Systems Informatics, Yonsei University College of Medicine, Seoul, Republic of Korea; ^7^Center for Digital Health, Yongin Severance Hospital, Yongin, Republic of Korea

**Keywords:** delirium, machine learning, vital signs, early diagnosis, ischemic stroke

## Abstract

**Introduction:**

Delirium, frequently experienced by ischemic stroke patients, is one of the most common neuropsychiatric syndromes reported in the Intensive Care Unit (ICU). Stroke patients with delirium have a high mortality rate and lengthy hospitalization. For these reasons, early diagnosis of delirium in the ICU is critical for better patient prognosis. Therefore, we developed and validated prediction models to classify the real-time delirium status in patients admitted to the ICU or Stroke Unit (SU) with ischemic stroke.

**Methods:**

A total of 84 delirium patients and 336 non-delirium patients in the ICU of Ajou University Hospital were included. The 8 fixed features [Age, Sex, Alcohol Intake, National Institute of Health Stroke Scale (NIHSS), HbA1c, Prothrombin time, D-dimer, and Hemoglobin] identified at admission and 12 dynamic features [Mean or Variability indexes calculated from Body Temperature (BT), Heart Rate (HR), Respiratory Rate (RR), Oxygen saturation (SpO2), Systolic Blood Pressure (SBP), and Diastolic Blood Pressure (DBP)] based on vital signs were used for developing prediction models using the ensemble method.

**Results:**

The Area Under the Receiver Operating Characteristic curve (AUROC) for delirium-state classification was 0.80. In simulation-based evaluation, AUROC was 0.71, and the predicted probability increased closer to the time of delirium occurrence. We observed that the patterns of dynamic features, including BT, SpO2, RR, and Heart Rate Variability (HRV) kept changing as the time points were getting closer to the delirium occurrence time. Therefore, the model that employed these patterns showed increasing prediction performance.

**Conclusion:**

Our model can predict the real-time possibility of delirium in patients with ischemic stroke and will be helpful to monitor high-risk patients.

## 1 Introduction

Delirium, an acute neuropsychiatric syndrome, is characterized by a sudden loss of attention, change in consciousness level, and fluctuating cognitive impairments, including memory dysfunction (Inouye et al., 2014). It occurs in one-third of the hospitalized older adults aged ≥70 years and more than half of the patients requiring mechanical ventilation (Marcantonio, 2017; Almeida et al., 2014). Clinically, delirium is an independent factor associated with poor outcomes in various study groups (Wilson et al., 2020) and is associated with higher mortality, longer hospital stay, worse functional outcomes, and sustained cognitive dysfunction even after discharge (Wilson et al., 2020; Pandharipande et al., 2013; Kosar et al., 2017; Burry et al., 2019).

Early diagnosis of delirium is important because timely administration of relevant drugs is critical for delirium management (Flinn et al., 2009; Barr et al., 2013). Recent studies showed that several pharmacological agents including haloperidol and nonpharmacological multi-component interventions could be useful for reducing the incidence, duration, recurrence, and mortality in ICU patients (Wang et al., 2012; van den Boogaard et al., 2013; Milbrandt et al., 2005; Schneider et al., 2005; Duan et al., 2018; Oliven et al., 2021; Rohatgi et al., 2019). However, delirium is often misdiagnosed as pain or depression and the subtle changes in the initial stages of delirium are overlooked (Inouye et al., 2001; de la Cruz et al., 2015; Han et al., 2009).

Several delirium assessment tools, rather than diagnostic criteria, have been developed for accessibility and episodic use when delirium is suspected or for regular monitoring of new-onset delirium (American Psychiatric Association D, Association AP, 2013; Sartorius et al., 1993). The assessment tools include the Confusion Assessment Method (CAM), the Confusion Assessment Method for the ICU (CAM-ICU), 4A’s Test (4AT), and Intensive Care Delirium Screening Checklist (ICDSC) (Inouye et al., 1990; Ely et al., 2001; Bellelli et al., 2014). Furthermore, prediction models including delirium assessment tools such as prediction diagnostic tools (PRE-DELERIC) and early predication model (E-PRE-DELERIC) have been developed for ICU patients (van den Boogaard et al., 2012; van den Boogaard et al., 2014; Wassenaar et al., 2015). In addition, the Lanzhou and the VR-PRE-DELIRIC models were developed using Machine Learning (ML) models based on prospective data. The R-PRE-DELIRIC model also proposed a prediction model using Logistic Regression (LR) (Green et al., 2019; van den Boogaard et al., 2014; Lee et al., 2017). However, these prediction models are clinically limited because they can predict occurrence of delirium at a specific time point such as admission.

The precise mechanism underlying the development of delirium is unclear. However, the postulated mechanisms include neurotransmitters, inflammation, physiological stressors, metabolic derangements, electrolyte disorders, and genetic factors (Inouye et al., 2014; Wilson et al., 2020). Further, evidence shows that delirium is associated with Autonomic Nervous System (ANS) instability (Liem and Carter, 1991; Jooyoung et al., 2017). A previous study has suggested that blood pressure and heart rate are related to autonomic function in patients with delirium (Oh et al., 2018).

In this study, we aimed to develop a novel ML model to predict occurrence of delirium using fixed and dynamic features associated with ANS, as ML techniques can be useful for analyzing complex signals in continuous data-rich environments such as ICUs. The proposed approach utilizes various methods to extract complex biosignal features for early detection and management of delirium in ICU settings. In addition, since the confounders of delirium are diverse and complex, we aimed to apply this model to a single neurological disease such as ischemic stroke to minimize the disease-related confounding variables. We also validate the applicability of ML-based delirium prediction models in clinical practice through simulation-based and temporal evaluation with clinical dashboard.

## 2 Materials and methods

In this section, we will experiment with various ML models using fixed features from Electronic Medical Records (EMR) and dynamic features from biosignals (i.e., vital signs) as well as the selection of important variables. Additionally, the final model will be validated using a simulation-based and temporal evaluation to verify its applicability in clinical settings.

### 2.1 Data sources

This study included patients with ischemic stroke admitted to the Neuro ICU (NCU) and SU of Ajou University Hospital. Patients with acute stroke are admitted either to the NCU or the SU. Patients admitted to the NCU typically have more severe strokes; admissions to the NCU or SU may also be based on room availability rather than stroke severity. Patients in the NCU are transferred to the SU once their condition stabilizes; however, close observation remains required. The incidence of delirium was 25% in the SU and 31% in the NCU. Data from July 2019 to December 2020 were utilized for model development and retrospective evaluation, while data from March 2023 to May 2023 were used for temporal evaluation.

### 2.2 Input variables

The fixed features at admission were based on the initial data collected at the time of admission, which were extracted from the EMR, and the dynamic features based on vital signs were extracted from the electronic medical records and patient monitoring device. The dynamic features comprised HR, RR, SpO2, DBP, and SBP, for which one measurement was taken every minute. BT was measured hourly. We used the dynamic features before event onset and fixed features obtained at ICU admission to reflect the precipitating and predisposing factors, respectively. All laboratory tests were performed within 24 h of hospitalization.

### 2.3 Primary outcome

**Figure 1:**
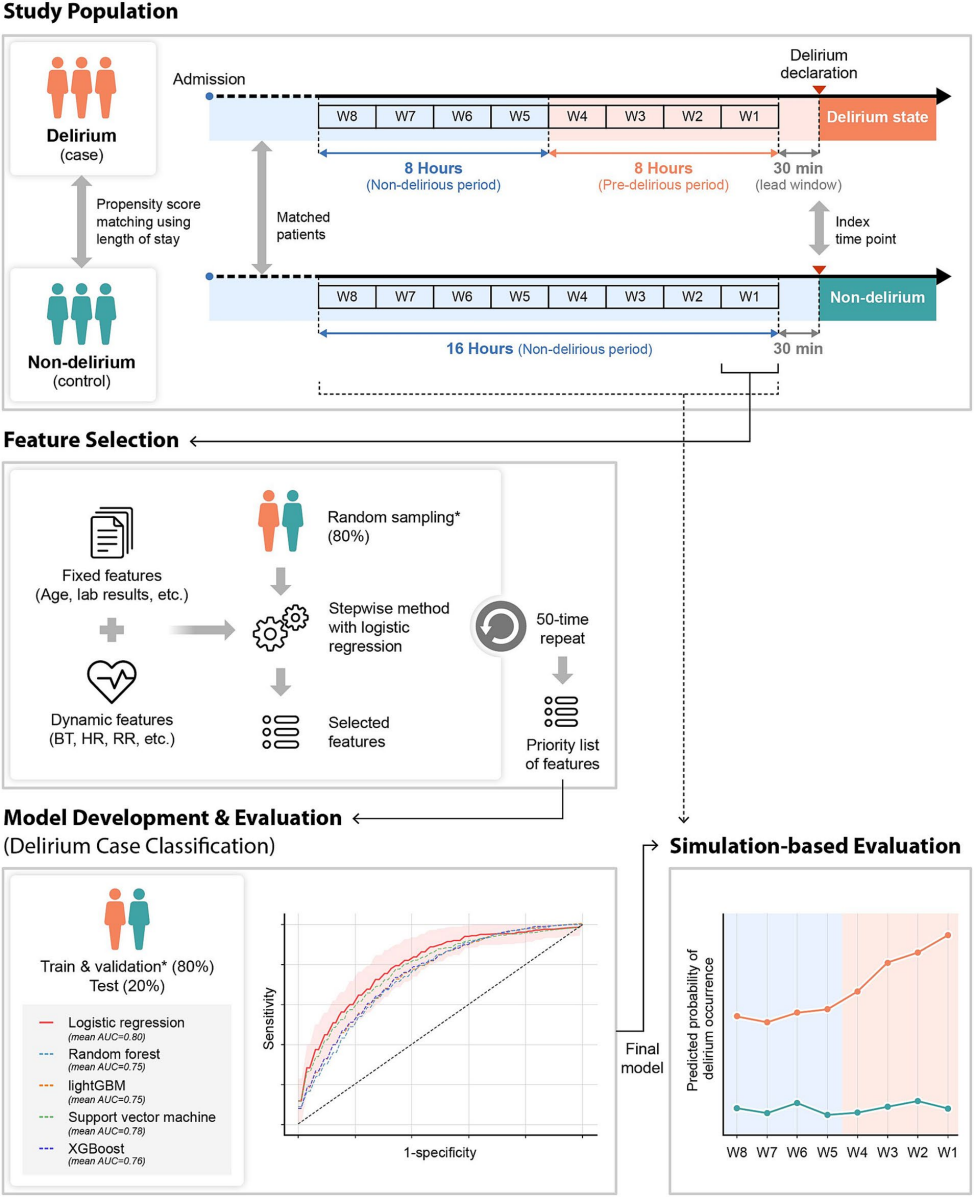
Overview of the study design. Fixed and dynamic features were used for the delirium prediction model development. For model development, dynamic features that were collected 2 h before the time point of delirium occurrence (case group) or matched time point (control group) were utilized. The stepwise method was used for feature selection. With the selected features, several machine learning algorithms were used and evaluated for model development. The final selected model was evaluated in a simulation environment by applying the model from 16 h before to the index time point every 2 h. W1–W8: the observation window 1–8; *the same dataset. BT, body temperature; HR, heart rate; RR, respiratory rate.

The study patients were selected and divided into case and control groups according to their delirium status based on the CAM-ICU or ICDSC assessed by the nurse at the NCU and SU. The CAM-ICU or ICDSC was used in combination with thorough chart reviews of clinical data and nursing records by two trained neurologists for the final subject selection. Patients positive on CAM-ICU or those with an ICDSC score of ≥4 were considered to have delirium at admission. The ICDSC was measured by a nurse periodically every 8 h and immediately at the NCU or SU if delirium was suspected. As the final step, a neurologist confirmed the diagnosis of delirium. For primary outcome labeling, we classified two groups: delirium and non-delirium. Subsequently, we used a 30-min period right before the event occurrence for prediction time for model development. Finally, we divided the 16-h period right before the occurrence of the event into 2-h observation windows. Then, we labeled four observation windows that were close to the event as positive for delirium status (pre-delirious period) and others as negative for non-delirium status (non-delirious period) for simulation-based evaluation, because the evaluation of delirium was conducted every 8 h usually (Figure 1).

### 2.4 Data preprocessing

#### 2.4.1 Dynamic feature extraction

All dynamic features were vital sign-based features, and they were extracted from the data of the 2-h observation period. The mean, Standard Deviation (SD) of vital signs were obtained from all 2-h observation periods. When we compared the statistical values between the case and control groups, BT and HR variables showed significant differences. However, basic statistical values like SD were not enough to capture the complex patterns in the biosignal data. As a result, we used advanced analytical methods to extract variability features. The time domain, and non-linearity were used for variability calculation. In the case of waveform data, frequency domain features were also extracted.

Dynamic features reflecting variability were calculated using Poincaré plot, Detrended Fluctuation Analysis (DFA), and sample entropy to capture short-and long-term variability, correlations, and irregularity in time-series data. Poincaré plot is a geometrical method of graphing each data point against the next data point, providing insight into patterns within the data measured over the short term (Karmakar et al., 2009; Woo et al., 1992). DFA measures the long-range correlation and dependencies of a time series over a range of time scales (Peng et al., 1994). Sample Entropy quantifies the complexity and measures the irregularity within time series data. Lower value means regularity, and higher value means irregularity (Yentes et al., 2019; Richman and Moorman, 2000). These methods are widely used in time-series analysis for physiological signals. Detailed methodologies for each approach can be found in Karmakar et al. (2009), Woo et al. (1992), Peng et al. (1994), Yentes et al. (2019), and Richman and Moorman (2000).

**Figure 2:**
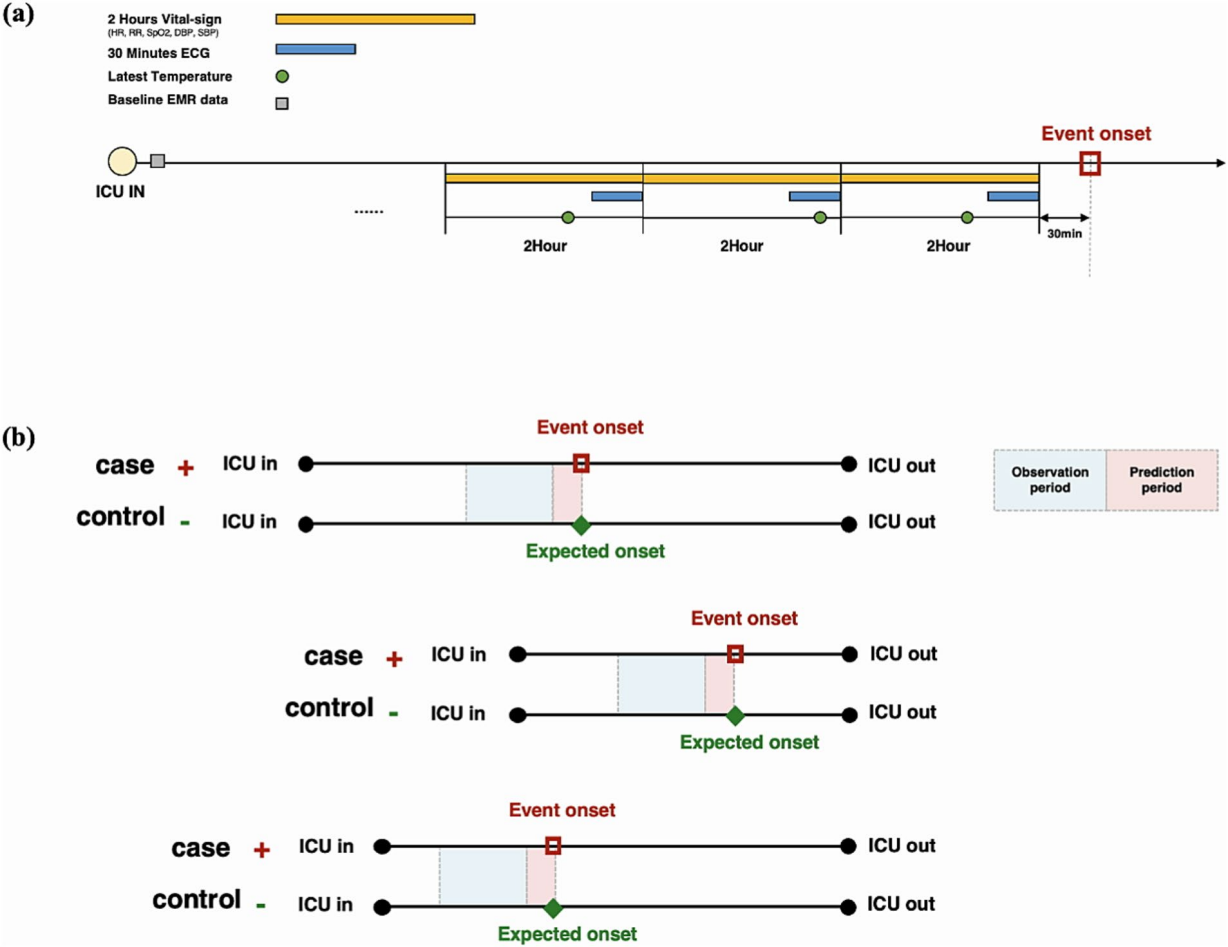
Input variable extraction strategy in each observation time window. **(A)** In addition to baseline data at admission, 2-h vital signs, the latest 30-min electrocardiogram, and body temperature measurement in each time window are used for model training. **(B)** All patients are matched by the length of stay in the intensive care unit using the propensity score matching method.

All variability metrics used are summarized in Supplementary Table S1. The electrocardiogram (ECG) waveform was collected in the most recent 30 min in each observation window, and the variability of respiratory rate interval was extracted through ECG that was down-sampled at 125 Hz (Figure 2).

#### 2.4.2 Imputation

The problem of missing values is common in clinical data, and the dataset of the model developed also had missing values. To solve this problem, we experimented using mean value replacement, median value replacement, and Multivariate Imputation by Chained Equations (MICE) replacement methods, and the MICE method with the best performance was finally adopted. The MICE algorithm can impute both continuous and categorical data, and it can simultaneously estimate missing values for multiple variables. This method not only considers the relationships between each variable but also performs multiple iterations of imputation. Details about the MICE methodology are in Azur et al. (2011).

### 2.5 Study design

**Figure 3:**
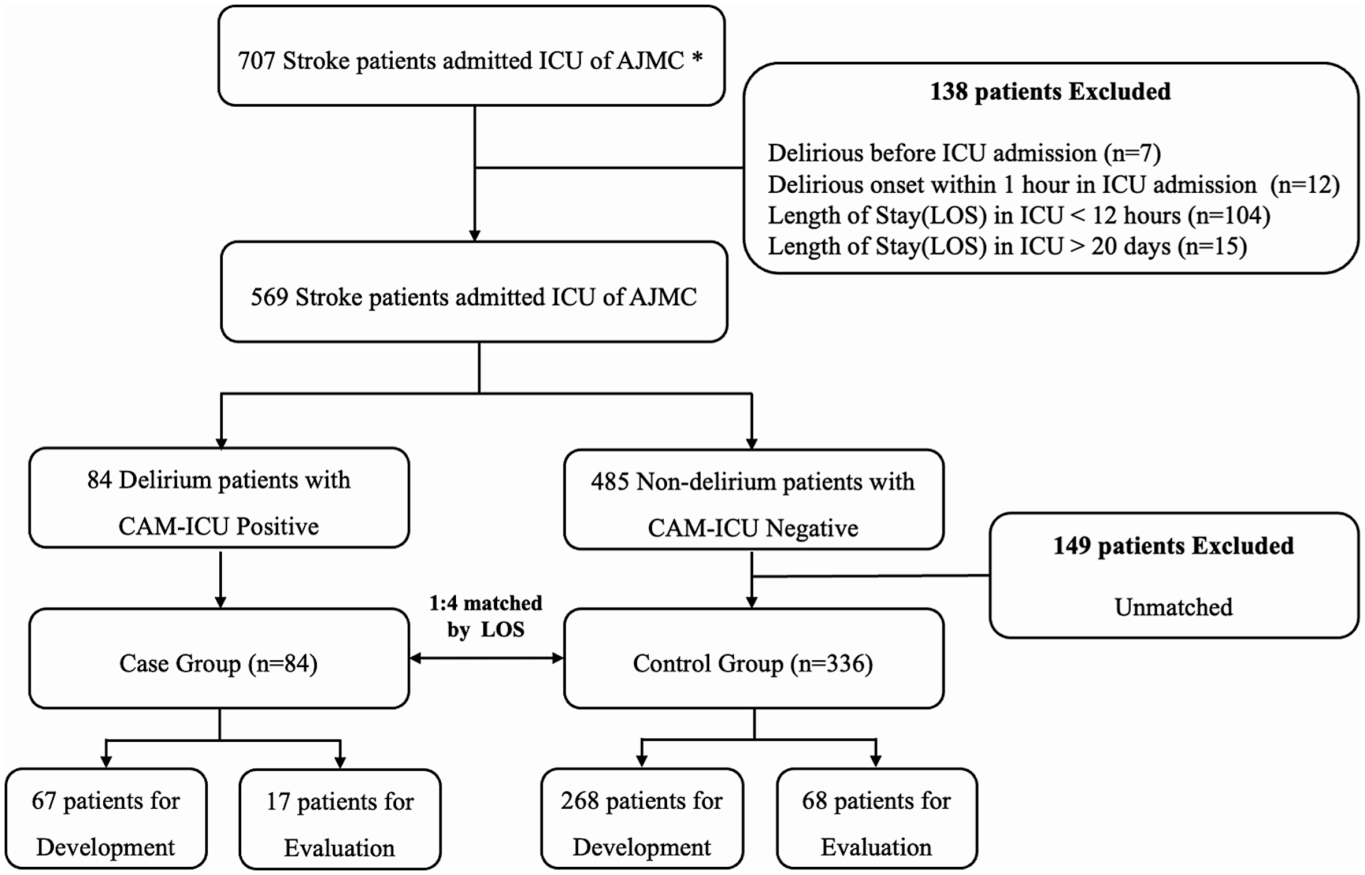
Inclusion and exclusion criteria for model development. All patients admitted to the intensive care unit (ICU) between July 2019 and December 2020 were enrolled. Two trained neurologists selected all patients by reviewing clinical data and nursing records. Finally, 67 patients with delirium (case group) and 268 patients without delirium (control group) were used for model development, and 90% were used for training and 10% for validation. In total, 17 patients with delirium and 68 patients without delirium were used for model evaluation. *AUMC, Ajou University Medical Center; CAM, confusion assessment method.

The study flow diagram is shown in Figure 3. Model development was based on a case–control study to analyze patients with and without delirium. The study included 707 patients with acute stroke admitted to the SU and NCU at Ajou University Hospital from July 2019 to December 2020. Among the 707 patients with stroke admitted to the ICU at Ajou University Hospital, 138 were excluded because delirium already occurred before admission to the ICU (*n* = 7), delirium occurred within 2 h of ICU admission (*n* = 12), and ICU Length Of Stay (LOS) was either too short (<12 h, *n* = 104) or too long (≥20 days, *n* = 15). The remaining 84 and 485 patients with and without delirium, respectively, were matched using the Propensity Score Matching (PSM) method. PSM is a method with LR to match two groups into a comparable state. Details has been described in Peter (2011). We used PSM to control confounding factors between delirious patients and non-delirious patients. The LOS variable was used to match patients with and without delirium (within 1-day difference). The LOS was chosen as the matching variable because it indicates patient severity, and the time of data extraction needs to be the same for patients with and without delirium. Therefore, the data could be extracted at similar time points for patients with and without delirium with a similar level of severity. The temporal validation cohort consisted of 149 patients admitted from January to May 2023; 115 were included after applying the exclusion criteria.

### 2.6 ML model

#### 2.6.1 Model development

**Figure 4:**
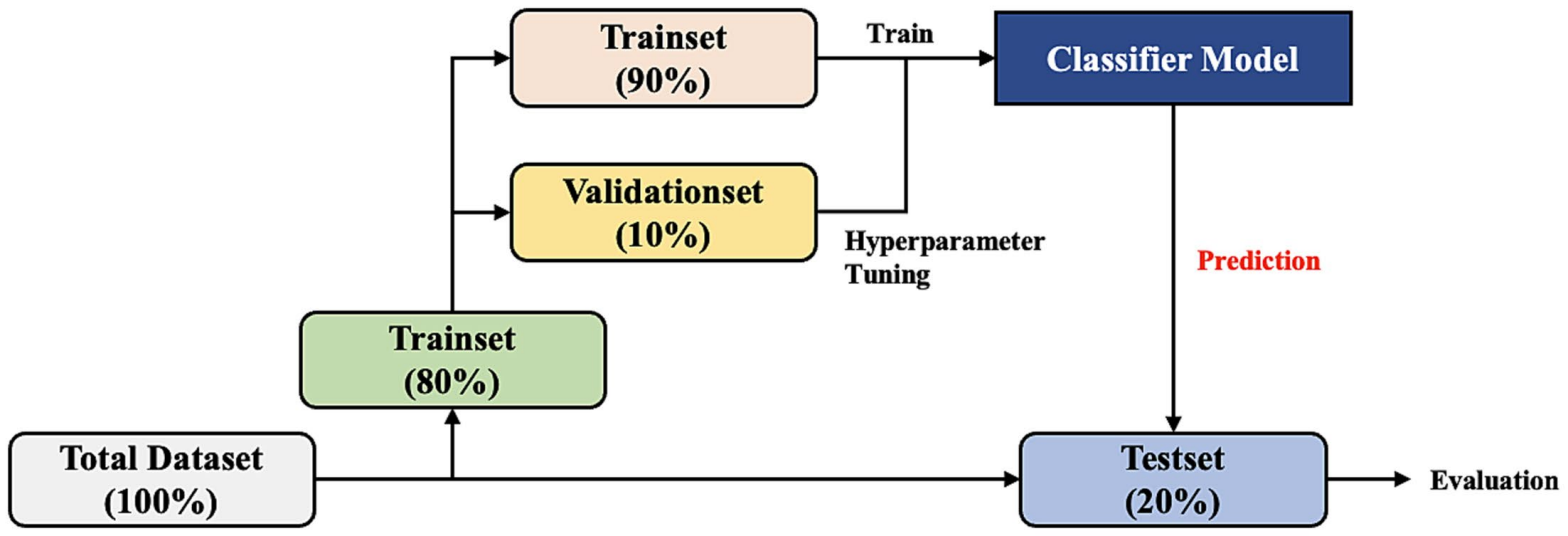
Dataset split workflow. The total dataset is divided by patient into the training set (80%) and test set (20%). Then, 10% of the training set is divided into the validation set for hyperparameter tuning of the model.

We developed the model to predict occurrence of delirium in the ICU in 420 patients with stroke. After data preprocessing and extraction, we split the entire dataset patient-wise. The case and control groups were randomly divided into the training set (80%) and test set (20%) and then stratified to unify the labeling ratio of each dataset (case:control = 1:4 for the training set and case:control = 1:4 for the test set). Then, 10% of the training set was divided into the validation set for hyperparameter tuning (Figure 4).

To measure the generalized model performance, the training, validation, and test datasets were randomly split 50 times. Finally, we prepared 50 datasets to train and evaluate the delirium prediction model.

We conducted feature reduction for removing model complexity. We extracted 109 features. To reduce unimportant features and overcome the limitations of a small dataset, we applied a stepwise statistical variable selection method to finalize the predictors. The stepwise selection method was used for each of the 50 different training sets described above. A variable that was selected many times during 50 iterations was listed as the higher priority. For example, the variable selected 50 times in all iterations was defined as the most important feature, and the variable selected 0 times in all iterations was defined as the most unimportant feature.

Stepwise variable selection using a *p*-value of 0.05 as an inclusion and exclusion threshold was performed for adding statistically significant variables and those that were not. By using this method, it enabled us to find the most informative features and reduce the complexity of the model that prevented overfitting. This not only helped the generalization of the model, especially with small datasets, but also reduced noise variance and protected the algorithm from overparameterization.

Owing to the limited number of data samples, we could not use a deep-learning model. Thus, we used machine-learning models. Most prior studies presented models using LR. In contrast, we used multiple models for performance comparison and analysis. The LR, Random Forest (RF), LightGBM (LGBM), Support Vector Machine (SVM), and XGBoost (XGB) were used for training the developed delirium prediction model. Each algorithm m was trained 50 times with 50 training datasets. We also developed and compared models using different number of features (top 10, top 20, top 30, top 40, top 50, and all features from the feature priority list).

#### 2.6.2 Classification evaluation

The performance of the model used to classify delirium status 30 min before the event onset was assessed. The mean performance and 95% CI for the 50 models developed were measured. The optimal cut-off was defined using Youden’s index in the validation dataset to set the cut-off of the model prediction. The Youden’s Index is a method to find the optimal threshold that maximizes sensitivity and specificity, which is useful even when the class is unbalanced (Fluss et al., 2005). The indicators of performance were AUROC, average area under the precision-recall curve (AUPRC), accuracy, precision, recall, F1 score, sensitivity, and specificity. Additionally, to verify the influence of the bio-signal data, the variables were categorized as follows for comparison: top 20 features among only fixed features, top 20 features among only dynamic features, and top 20 features among both fixed and dynamic features.

#### 2.6.3 Simulation-based evaluation

To apply the model in the real clinical environment, the model needs to predict continuously from the time patients are admitted to the ICU. For this simulation-based evaluation, we applied the trained model to evaluate in test set using all observational windows defined in Figure 1 (i.e., window1–8). Then, we confirmed the trend of the model’s prediction probability during 16 h before event occurrence in every 2-h time window. This simulation based evaluation approach can reflect clinical workflows and make timely predictions to help clinicians and nurses make clinical decisions based on real-time data.

#### 2.6.4 Temporal evaluation

**Figure 5:**
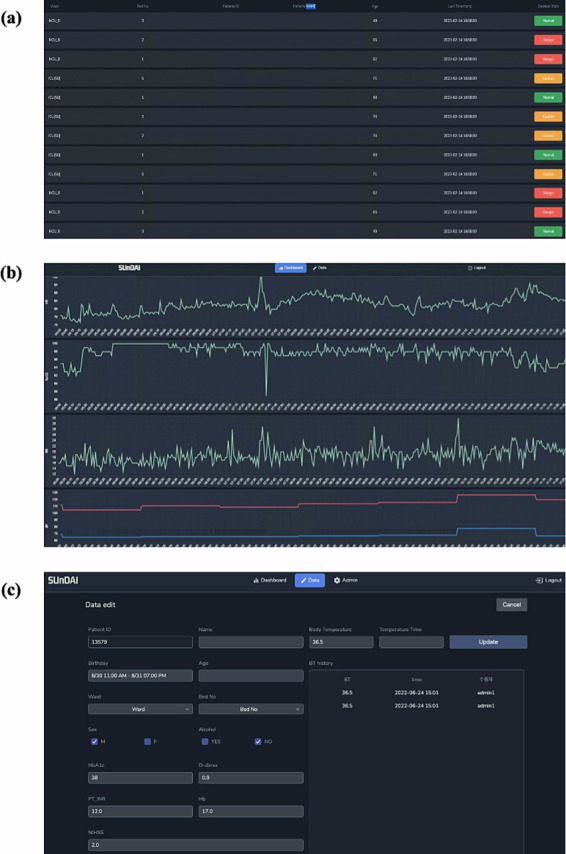
Delirium occurrence alarm system embedded in the prediction model. This image shows the clinical dashboard user interface used for temporal validation. **(A)** Alarm Dashboard page, **(B)** Model Prediction and vital sign trend view page, and **(C)** Data input page.

We validated our model temporally using a specialized user interface designed for this task (Figure 5). This model was employed for all stroke patients admitted to the SU and the NCU from March 1 through May 3, 2023. Temporal validation process was executed in three stages. First, the discriminative capability of the model was evaluated by differentiating the onset of delirium (using data from within 2 h before delirium event documentation) from a matched non-delirium group. Next, the model’s ability to forecast the risk of delirium onset during the 16 h leading up to a delirium event was compared. Within this period, the 8 h immediately before the 16 h of delirium were explicitly designated as the “pre-delirious period,” consistent with our retrospective validation method. These first two stages adopted the same approach used in our retrospective validation. Finally, the model was applied throughout the patient’s ICU stay. An 8-h “pre-delirious period” before any confirmed delirium event was set for detection. Observations were ended at the time of the first confirmed delirium event or upon the patient’s discharge.

#### 2.6.5 Statistical analysis

Univariate analysis was performed to statistically compare independent variables between the case and control groups. For continuous data such as HR and SpO2, the between-group variation was analyzed using the two-sample two-tailed *t*-test. For categorical data such as sex, smoking, and alcohol drinking, the chi-squared test was used. The level of significance was set at *p* < 0.05.

#### 2.6.6 Software

All analyses were performed using Python version 3.7, the Python package scikit learn 1.0.1, pandas 1.1.5, numpy 1.19.5, scipy 1.4.1, and pyhrv 0.4.0. The Python packages Matplotlib 3.2.2 and Seaborn 0.11.2 were also used to visualize the data and results.

## 3 Results

### 3.1 Baseline characteristics

The baseline characteristics of the case and control groups are presented in Supplementary Table S2. The NIHSS score indicating stroke severity (*p* = 0.016), premorbid modified Rankin Scale (mRS) score (*p* = 0.013), and number of older patients (*p* < 0.001) were higher in the case group than in the control group. In addition, the HR (*p* = 0.023), RR (*p* < 0.001) and BT (*p* < 0.001) were significantly higher in the case group than in the control group. The variability features are shown in Supplementary Table S3.

### 3.2 Models for prediction of delirium occurrence

**Figure 6:**
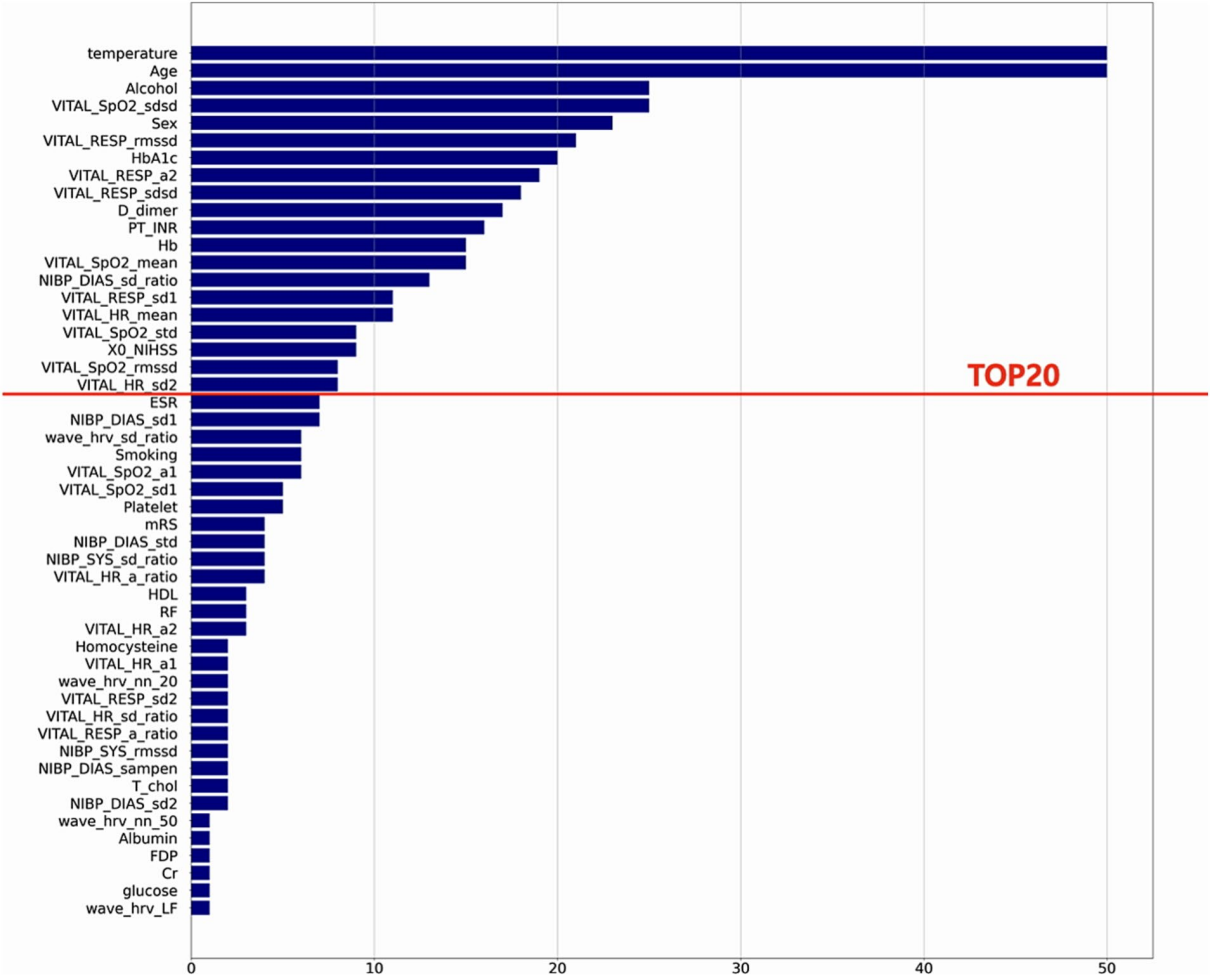
Feature selection results. This plot shows the final variable list selected through stepwise feature selection method. The final ML model included top 20 features.

The result of variable selection across all variables is shown in Figure 6. The top 50 variables were evaluated for model development: 17 variables were from the fixed features (clinical features at admission) and 33 variables from the dynamic features (features based on vital signs). Finally, 20 variables—8 variables from the fixed features and 12 variables from the dynamic features—were selected for the final model (Table 1).

**Table 1:** Baseline Characteristics of the study population (Top 20 features).

Variable	Case (*n* = 84)	Control (*n* = 336)	*p*-value	Selected for top 20 features
Demographics				
Age, year	73.81 ± 11.24	63.6 ± 14.04	<0.001*	√
Sex (male), %	64.28	61.6	0.744	√
Alcohol, %	52.38	42.55	0.134	√
Fixed features				
NIHSS	7.98 ± 5.2	6.25 ± 5.78	0.016*	√
HbA1c, %	6.66 ± 1.72	6.27 ± 1.41	0.032*	√
Prothrombin time (INR)	1.58 ± 2.65	1.22 ± 1.34	0.081	√
D-dimer, ug/mL	2.79 ± 6.34	1.29 ± 3.36	0.003*	√
Hemoglobin	15.99 ± 25.95	13.8 ± 2.32	0.131	√
Dynamic features				
BT, °C	36.97 ± 0.42	36.79 ± 0.43	<0.001*	√
HR, bpm	75.49 ± 15.18	71.22 ± 14.36	0.023*	√ (mean, SD2)
RR, breaths/min	18.21 ± 2.99	17.59 ± 3.1	0.114	√ (a2, SD1, SDSD, RMSSD)
SpO2, %	96.64 ± 1.39	96.23 ± 3.19	0.271	√ (mean, SDSD, SD, RMSSD)
SBP, mmHg	147.91 ± 21.84	147.04 ± 23.47	0.770	
DBP, mmHg	84.97 ± 13.63	86.07 ± 13.37	0.149	√ (SD ratio)

*p < 0.05. ^**†**^Number of comorbidities among hypertension, dyslipidemia, or diabetes; NIHSS, National Institute of Health Stroke Scale; mRS, modified Rankin Scale; SD1/SD2, Poincaré plot standard deviation of the major/minor axis; SDSD, standard deviation of successive difference; RMSSD, root mean square of successive difference; SD, standard deviation; SD ratio, ratio between SD1 and SD2 (SD2/SD1).

### 3.3 Model performance

#### 3.3.1 Evaluation of delirium event classification

**Figure 7:**
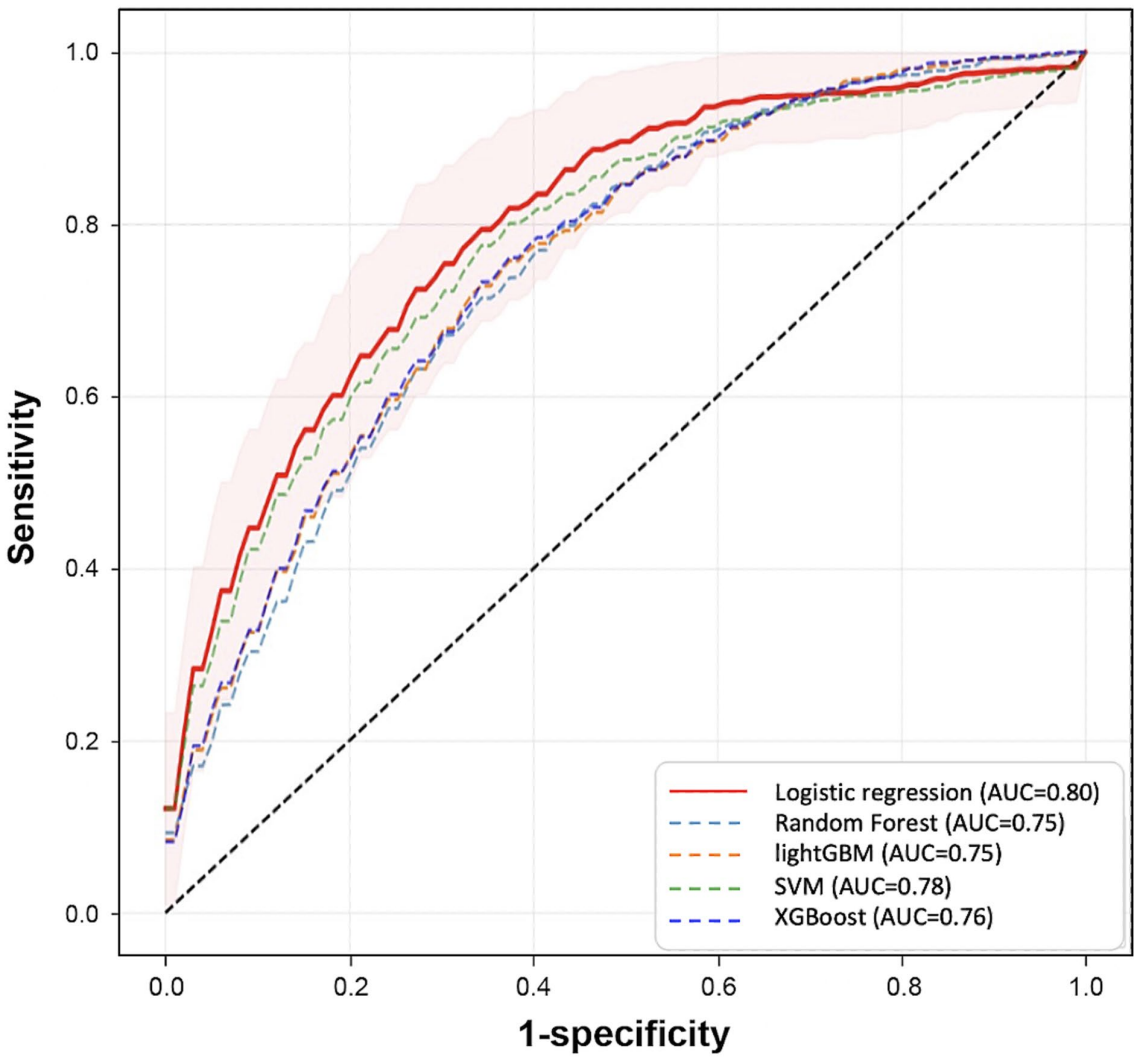
Comparison of prediction performance across different Machine Learning algorithms. This plot shows the Receiver Operating Characteristic curves of each model that is trained by different algorithms (LR, RF, SVM, LGBM, XGB) using the top 20 variables. These results are calculated from the mean performances of 50 models trained by different randomly selected training datasets. The red area represents the mean ± SD of the performance of the final model.

Among ML algorithms, LR showed the highest performance in delirium event classification when the top 20 features were used (Table 2 and Figure 7). Our model showed an AUROC of 0.80 (95% confidence interval [CI]: 0.78–0.81) and an AUPRC of 0.552 (95% CI: 0.525–0.579), and the model could classify delirium and non-delirium status with a sensitivity and specificity of 0.75 and 0.72, respectively.

**Table 2:** Model performance based on the feature set.

Features	AUROC (95% CI)	AUPRC (95% CI)	Precision (95% CI)	Recall (95% CI)	F1-score (95% CI)
Discrimination performance between delirium and non-delirium according to feature sets (retrospective evaluation)					
All feature	0.68 (0.67–0.7)	0.39 (0.36–0.41)	0.35 (0.32–0.38)	0.66 (0.62–0.71)	0.43 (0.42–0.45)
Top 50 features	0.72 (0.7–0.74)	0.45 (0.43–0.48)	0.41 (0.38–0.44)	0.64 (0.6–0.69)	0.48 (0.46–0.49)
Top 40 features	0.75 (0.73–0.76)	0.49 (0.46–0.51)	0.4 (0.38–0.43)	0.7 (0.66–0.74)	0.5 (0.48–0.51)
Top 30 features	0.79 (0.77–0.8)	0.53 (0.51–0.56)	0.43 (0.41–0.46)	0.73 (0.7–0.77)	0.53 (0.51–0.55)
Top 20 features	0.80 (0.78–0.81)	0.55 (0.52–0.58)	0.42 (0.4–0.44)	0.75 (0.72–0.79)	0.53 (0.51–0.55)
Top 10 features	0.74 (0.72–0.76)	0.47 (0.44–0.49)	0.42 (0.4–0.45)	0.66 (0.62–0.69)	0.49 (0.47–0.51)
Simulation-based evaluation performance (retrospective evaluation)					
Top 20 features	0.71 (0.7–0.73)	0.29 (0.27–0.31)	0.25 (0.23–0.26)	0.68 (0.63–0.72)	0.35 (0.33–0.37)
Discrimination performance between delirium and non-delirium (temporal evaluation)					
Top 20 features	0.78	0.49	0.41	0.62	0.50
Simulation-based evaluation performance (temporal evaluation)					
Top 20 features	0.70	0.31	0.27	0.61	0.37
Performance in entire ICU staying (temporal evaluation)					
Top 20 features	0.68	0.12	0.10	0.61	0.17

**Figure 8:**
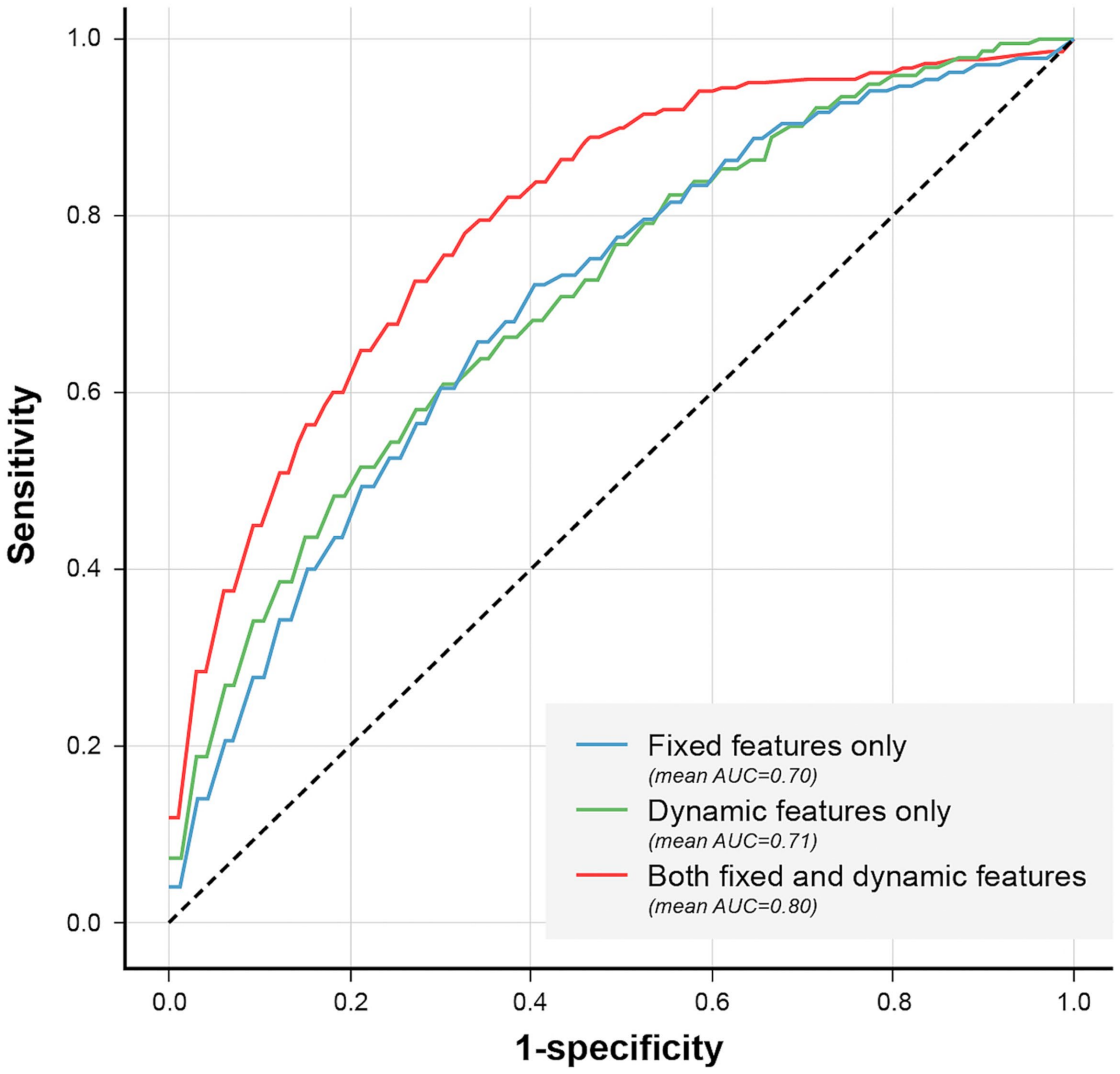
Comparison of models’ performance according to the used features. Comparison of models’ performance according to the used features. The AUROC was higher (0.80) when fixed and dynamic features were used together, compared to the models that used only fixed (0.70) or dynamic features (0.71).

We also compared the models when only fixed features were used and when the dynamic features were used together. When comparing the performance in terms of the different sources of features, the best performance was obtained when fixed and dynamic features were used together (AUROC: 0.8 [95% CI: 0.78–0.814]) (Figure 8 and Supplementary Tables S4, S5), followed by when only fixed features were used (AUROC: 0.701 [95% CI: 0.68–0.721]). The lowest performance was obtained when only dynamic features were used (AUROC: 0.68).

#### 3.3.2 Simulation-based evaluation

In simulation-based evaluation, all observation windows shown in Figure 1 were used for evaluation. The average of performance indices according to outputs of finally selected models (50 models trained by LR with the top 20 variables) was used. The performance at all time points showed an average AUROC and AUPRC of 0.71 and 0.29, respectively (Table 2).

**Figure 9:**
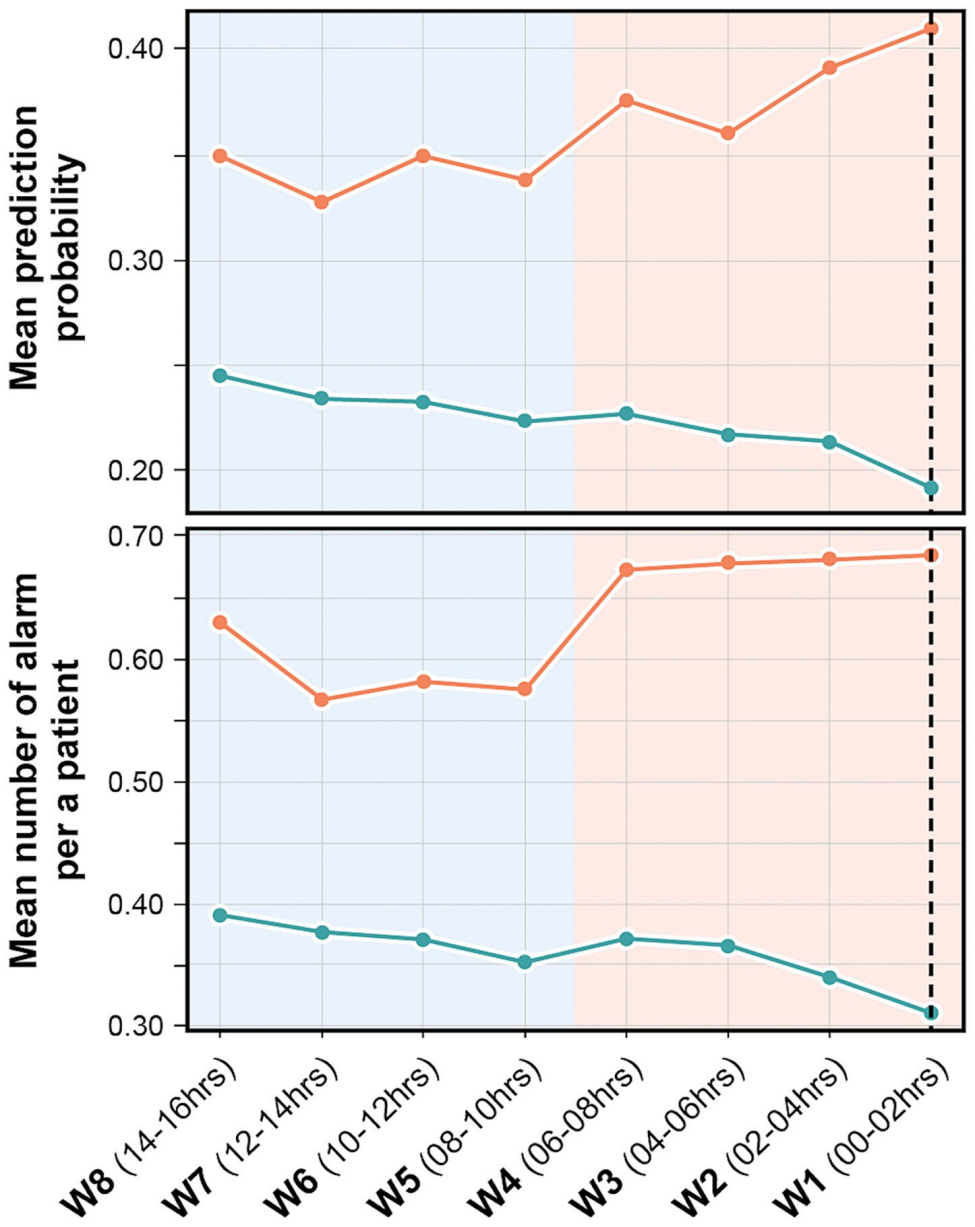
Mean prediction probability and mean number of alarms per patient in the retrospective validation. It shows the mean prediction probability of the model by time point (above), and the number of alarms per patient by time point (below). The orange line indicates delirium group and green line indicates non-delirium group. The black dashed line indicates the time point before 30 min of actual delirium event occurrence. The model’s prediction probability is higher and higher recall performance is seen closer to the event occurrence.

As shown in Figure 9, the model’s mean prediction probability by time point increased as the time points approached the event onset in the case group (orange line). In addition, the mean number of alarms per patient by time point was higher for patients with delirium than for patients without delirium and as the time approached the event onset (Figure 9). The models’ mean prediction probability and total number of alarms per patient in this simulation are presented in Supplementary Table S6.

#### 3.3.3 Temporal evaluation

**Figure 10:**
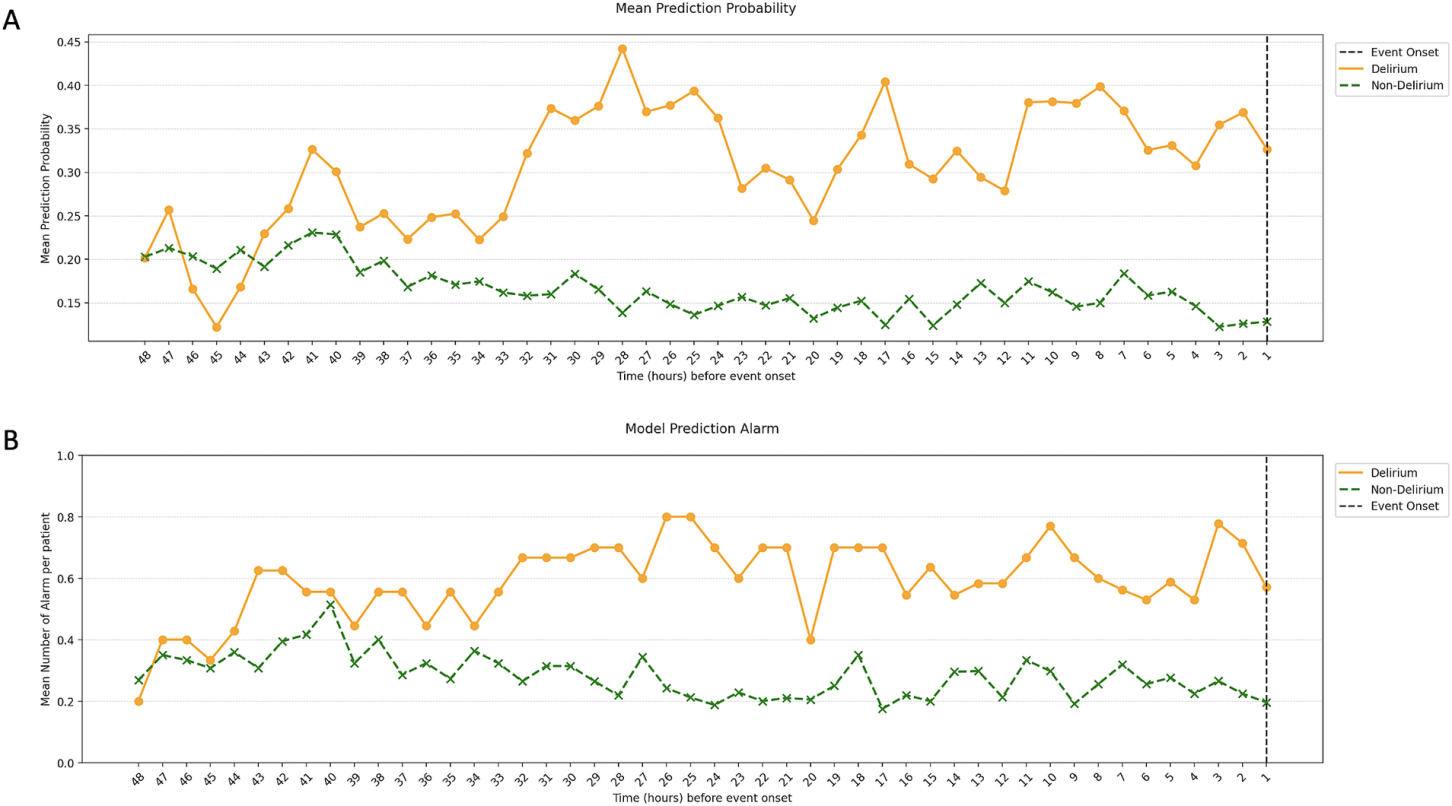
Mean prediction probability and mean number of alarms per patient in the temporal validation. The image shows the mean prediction probability of the model by time point **(A)** and the number of alarms per patient by time point **(B)**. Similar to the retrospective validation, we saw a gradual increase in prediction probability and the number of alarms closer to the time of the event, with this pattern starting around 48 h before the delirium.

The results of our model’s temporal validation are closely aligned with those of the retrospective evaluation. When assessing delirium occurrence against the matched non-delirium group, the AUROC was 0.78, and the AUPRC was 0.49. In the 16 h leading up to delirium, these values were 0.70 and 0.31, respectively (Table 2). Evaluating over the entire duration of the ICU stay yielded an AUROC of 0.68 and an AUPRC of 0.12, illustrating a decrease in precision and lower AUPRC values as the assessment period lengthened. However, a positive correlation was observed between the approach of a delirium event, the delirium probability value, and the frequency of alarms (Figure 10). Consequently, despite false alarms, it can be inferred that these alerts were meaningful due to their temporal proximity to delirium events.

#### 3.3.4 Predicted risk of delirium and patient outcomes

We examined the relationship between the predicted risk of delirium and the actual outcomes of patients evaluated during retrospective and temporal validation. When comparing high-risk patients (mean predicted risk of delirium ≥0.2) and low-risk patients (mean predicted risk of delirium <0.2) during the retrospective validation, the ICU length of stay and 3-month mRS score were statistically significantly higher in the high-risk group (5.57 days, *p* = 0.014 and 2.98, *p* < 0.001, respectively) than the low-risk group (3.98 days and 1.39). These trends were also observed in the temporal validation; high-risk patients with a mean risk of delirium ≥0.2 had a mean ICU length of stay of 5.8 days (*p* = 0.001), compared with 3.27 days for low-risk patients. This difference was also observed when the cut-off for separating the high-risk and low-risk groups was varied (Table 3).

**Table 3:** Comparison of clinical outcomes in high-and low-risk groups.

Cut-off	Risk group	ICU LOS (days)	*p*-value	3 month mRS	*p*-value
Retrospective					
0.2	High	5.57 ± 8.84	0.014	2.98 ± 1.82	< 0.001
	Low	3.98 ± 4.41		1.39 ± 1.5	
0.3	High	6.2 ± 10.16	0.001	3.06 ± 1.71	< 0.001
	Low	3.97 ± 4.24		1.57 ± 1.65	
0.4	High	6.06 ± 8.29	0.023	3.05 ± 1.73	< 0.001
	Low	4.21 ± 5.82		1.71 ± 1.71	
Temporal					
0.2	High	5.8 ± 4.88	0.001	–	–
	Low	3.27 ± 2.92		–	–
0.3	High	6.26 ± 4.08	0.005	–	–
	Low	3.68 ± 3.64		–	–
0.4	High	6.73 ± 4.09	0.005 *	–	–
	Low	3.68 ± 3.64		–	–

*p < 0.05.

## 4 Discussion

Our prediction model using ML could predict the occurrence of delirium, and the model’s predictive probability of delirium significantly improved closer to the time of delirium occurrence. In our study, we conducted a temporal evaluation of our model’s performance. The findings revealed a consistency in the model’s performance, mirroring its effectiveness during the training phase. This alignment strengthens our confidence in the model’s robustness and potential as a practical tool in a clinical setting. Given its consistent performance, we anticipate that this model will serve as a valuable addition to clinical practice, aiding healthcare professionals in improving patient outcomes.

To predict the occurrence of delirium, it may be useful to use variable vital signs rather than identify the conventional risk factors of delirium, such as patient demographics and initial laboratory findings. Previous PRE-DELERIC and E-PRE-DELERIC models were static models that yield calculated probabilities of delirium 24 h after ICU admission (van den Boogaard et al., 2012; van den Boogaard et al., 2014). In another previous study, the heart rate of patients with delirium was more variable and irregular than that of patients without delirium (Jooyoung et al., 2017). In addition, a pilot study showed that in recent cases of delirium, blood pressure changed more significantly in the head-up tilt tests and that dynamic parameters were used to correlate with excessive sympathetic responses showing delirium predictability (Shanahan et al., 2021). Although delirium prediction models using ML have been developed, they have not continuously monitored the occurrence of delirium (van den Boogaard et al., 2012; van den Boogaard et al., 2014; Wassenaar et al., 2015; Bishara et al., 2022). One of the primary benefits of utilizing vital sign data is its potential for real-time delirium risk assessment, a marked improvement compared to existing models such as PREDELIRIC and E-DELIRIC. For validation, we compared our machine-learning model with the well-known delirium prediction tools, PRE-DELIRIC and E-DELIRIC. The cut-off was set identically to our model’s optimal threshold for comparing performance metrics. The model we developed demonstrated superior performance across all metrics, proving its clinical application is more effective than existing tools, which can only predict within 24 h of admission (Table 4).

**Table 4:** Comparison of the performances of PRE-DELIRIC and E-DELIRIC.

Model	AUROC (95% CI)	AUPRC (95% CI)	Precision (95% CI)	Recall (95% CI)	F1-score (95% CI)
PRE-DELIRIC	0.73	0.27	0.35	0.48	0.40
E-DELIRIC	0.72	0.28	0.34	0.56	0.42
Ours	0.80	0.55	0.42	0.75	0.53

The average AUROC of the model using fixed features alone was 0.70 (95% CI: 0.68–0.72); however, it increased to 0.80 (95% CI: 0.78–0.81) when the dynamic features were also used. This show that dynamic features can not only improve the performance of delirium prediction models but also be useful for predicting real-time delirium probability (Supplementary Table S5). In simulation-based evaluation, the model’s mean prediction probability by time point was higher for predicting delirium status closer to the event onset (Figure 9). Since continuous dynamic data were used, we could continuously predict the likelihood of delirium occurrence.

Despite the lack of clear evidence on drugs that can prevent delirium, a study has shown that short-term prophylactic administration of low-dose haloperidol is effective in preventing delirium in elderly ICU patients after noncardiac surgery (Wang et al., 2012). Appropriate administration timing and patient selection are required to effectively prevent delirium. Therefore, prediction and early detection of delirium using our model can help prevent delirium and reduce unnecessary utilization of medical resources as well as burden on medical staff, caregivers, and patients. Our research may facilitate the earlier prediction and detection of delirium, improving its prevention and treatment.

The 20 variables selected for ML in our study were consistent with the commonly known risk factors for delirium. Old age and diabetes are well-known risk factors for delirium, and stroke severity according to the NIHSS is also known as a risk factor for delirium (Oldenbeuving et al., 2011). In our statistical analysis, the NIHSS score showed significant differences between the two groups. This finding could be interpreted as delirium occurring due to a worsening stroke; however, we used the NIHSS score recorded at admission, which does not consider stroke deterioration, suggesting that the initial NIHSS score is important for classifying delirium. Therefore, the variables determined through ML are related to delirium. The trends in vital sign-based data allow us to gain insight into how the model detects changes in dynamic features before the delirium event (Figure 10). Characteristics such as BT and HRV showed more dynamic changes closer to the delirium onset. The BT of patients with delirium at baseline was higher at the baseline and lower than that before the delirium onset. HRV was similar between patients with and without delirium, but the variability increased before delirium occurrence. Moreover, SpO2 and RR variability were different between patients with and without delirium. Therefore, our study suggests that these dynamic changes controlled by the ANS may help distinguishing the two groups.

Our findings showed dynamic changes in the ANS in patients with delirium, which is consistent with the pathophysiology of delirium revealed in a previous study, indicating dysregulation of HRV and sympathetic hyperactivity. Several studies have suggested that an HRV alteration may help identify patients with stroke at risk for delirium in the ICU (Rollo et al., 2022). In addition, previous studies have suggested that delirium is associated with impaired oxidative metabolism (Taccone et al., 2010; Wood et al., 2017). A near-infrared spectroscopy study showed that low brain tissue oxygenation was an independent risk factor for delirium (Wood et al., 2017). Conversely, some studies have reported that perioperative hyperoxia may contribute to postoperative delirium (Lopez et al., 2017; Kupiec et al., 2020). Among the dynamic features considered in our study, SpO2 was higher in the case group than in the control group and decreased just before delirium occurrence. We believe that delirium is not associated only with tissue oxygenation, and our findings can provide a clue for connecting the contradictory findings on tissue oxygenation. One study had showed that temperature variability increased during delirium (van der Kooi et al., 2013).

In our study, we conducted a temporal application of our model, observing a performance largely congruent with our retrospective evaluation. Notably, when the model was utilized throughout the entire study period, we noticed a substantial decrease in precision as the number of predictions and non-delirium data escalated. However, a trend emerged showing an escalating proportion of alarms as the onset of delirium approached. This pattern could serve as a valuable guide, enabling clinicians to adjust their focus accordingly. In temporal application, we observed that the alerts made clinicians more focused on the onset of delirium in their patients, allowing them to identify new delirium cases quickly. Furthermore, patients exhibiting a higher predicted risk of delirium were found to have an increased 3-month mRS and longer ICU stays. However, the mortality rates of the case (7.14%) and control (6.25%) groups were not significantly different (*p* = 0.9603).

Though our temporal evaluation did not incorporate interventions, the significant difference in clinical outcomes within the high-risk delirium group provides a compelling rationale for the initiation of future studies that focus on early intervention strategies leveraging our predictive system.

However, our study has several limitations. First, since this model was only applied to patients with stroke, assessed using the NIHSS, it may be difficult to generalize this model to patients admitted to the ICU for other diseases. However, these shortcomings can be overcome using general scales representing the severity of each disease. It can be replaced by the Acute Physiology and Chronic Health Evaluation (APACHE) III or Sequential Organ Failure Assessment (SOFA) score, which are commonly used in the ICU (van den Boogaard et al., 2012; van den Boogaard et al., 2014). In addition, we focused specifically on stroke patients and included hypertension, diabetes, and dyslipidemia as stroke-related comorbidities. Nonetheless, incorporating well-known delirium risk factors, such as dementia and organ failure, can improve the performance of prediction models.

Second, during the temporal validation, we only observed the performance of the alarms and did not add clinical interventions based on the alarms. However, given that this study confirmed that the model-predicted risk levels and alarms were associated with adverse clinical outcomes, it may be possible to study the effectiveness of interventions using this alarm system in the future.

Third, our models have difficulty presenting a predictive score without patient monitoring. However, as the patients targeted in our study (those admitted to the NCU and SU) were fundamentally under vital sign monitoring, we believe this does not pose a significant problem.

Finally, this study did not conduct external validation. However, we collected data from different time points for further validation than the cohort used in the development. We also performed simulation validation, which indicated the evaluation is sufficiently reliable.

## 5 Conclusion

We believe that a prediction model using ML can provide timely prediction of the possibility of delirium in patients with ischemic stroke. In addition, the study revealed that vital-sign-based dynamic information is valuable for monitoring the risk of delirium occurrence.

## Data Availability

The data analyzed in this study is subject to the following licenses/restrictions: data cannot be shared publicly due to regulations from the Institutional Review Board of Ajou University Medical Center. This is because the data contain potentially identifying or sensitive patient information, and distributing these data could breach patient confidentiality. Data are available from the aforementioned Institutional Review Boards for researchers who meet the criteria for access to confidential data. Requests to access these datasets should be directed to ajouirb@aumc.ac.kr.
