# Supplementary material for: Prediction of delirium occurrence using machine learning in acute stroke patients in intensive care unit
Source: Front Neurosci. 2025 Jan 9;18:1425562. doi: 10.3389/fnins.2024.1425562 (PMC11754397; doi:10.3389/fnins.2024.1425562)
Supplement: Supplementary file 1 [file Table_1.DOCX]

Supplementary Material

**Supplementary Table 1.**

| **Metrics** | | **Definition** |
| --- | --- | --- |
| **General** | |  |
| Mean (only used for numerical data) | | Mean value of total data |
| Std (only used for numerical data) | | Standard deviation value of total data |
| **Time-domain** | |  |
|  | SDSD | Standard deviation of successive difference |
|  | RMSSD | Root mean square of successive difference |
|  | SDNN | Standard deviation of RR intervals |
|  | NN50 | The number of adjacent RR intervals that differ from each other by more than 50 ms |
|  | pNN50 | Percentage of successive RR intervals that differ by more than 50 ms |
|  | NN20 | The number of adjacent RR intervals that differ from each other by more than 20 ms |
|  | pNN20 | Percentage of successive RR intervals that differ by more than 20 ms |
| **Non-linear** | |  |
|  | **Poincare plot analysis** |  |
|  | SD1 | Poincare plot standard deviation (SD1) of the major axis |
|  | SD2 | Poincare plot standard deviation (SD2) of the minor axis |
|  | SD ratio | Ratio between SD1 and SD2 (SD2/SD1) |
|  | **Detrended fluctuation analysis** |  |
|  | a1 (Alpha 1) | Value of the short-term fluctuations |
|  | a2 (Alpha 2) | Value of the long-term fluctuations |
|  | a ratio (Alpha ratio) | Ratio between Alpha 1 and Alpha 2 (Alpha 2/Alpha 1) |
|  | **Sample entropy analysis** |  |
|  | SampEn | The regularity and complexity of a time series |
| **Frequency-domain** | |  |
|  | LVF | Relative power of the very low-frequency band (0.0033-0.04 Hz) |
|  | LF | Relative power of the low-frequency band (0.04-0.15 Hz) |
|  | HF | Relative power of the high-frequency band (0.15-0.4 Hz) |
|  | LF/HF | Ratio of LF-to-HF power |

SD1/SD2, Poincaré plot standard deviation of the major/minor axis; SDSD, standard deviation of successive difference; RMSSD, root mean square of successive difference

**Supplementary Table 2. Baseline characteristics of the study population (top 20 features)**

| **Variable** | **Case group**  **(n = 84)** | **Control group**  **(n = 336)** | ***p*-value** | **Selected for top 20 features** |
| --- | --- | --- | --- | --- |
| **Demographics** |  |  |  |  |
| Age, year | 73.81 ± 11.24 | 63.6 ± 14.04 | < 0.001* | √ |
| Sex (male), % | 64.28 | 61.6 | 0.744 | √ |
| Smoking, % | 54.76 | 50.0 | 0.510 |  |
| Alcohol, % | 52.38 | 42.55 | 0.134 | √ |
| Length of stay, days | 5.18 ± 7.06 | 4.37 ± 6.16 | 0.298 |  |
| **Fixed features at admission** |  |  |  |  |
| Number of comorbidities†, n | 1.63 ± 1.08 | 1.39 ± 1.04 | 0.063 |  |
| mRS (before admission) | 0.74 ± 0.94 | 0.49 ± 0.77 | 0.013* |  |
| NIHSS | 7.98 ± 5.2 | 6.25 ± 5.78 | 0.016* | √ |
| HbA1c, % | 6.66 ± 1.72 | 6.27 ± 1.41 | 0.032* | √ |
| Prothrombin time (INR) | 1.58 ± 2.65 | 1.22 ± 1.34 | 0.081 | √ |
| Partial thromboplastin time, second | 34.59 ± 6.32 | 34.14 ± 7.47 | 0.616 |  |
| D-dimer, µg/mL | 2.79 ± 6.34 | 1.29 ± 3.36 | 0.003* | √ |
| Fibrinogen degradation production, µg/mL | 9.99 ± 18.77 | 6.01 ± 12.29 | 0.020* |  |
| Fibrinogen, mg/dl | 337.6 ± 113.78 | 341.73 ± 195.51 | 0.856 |  |
| Hemoglobin | 15.99 ± 25.95 | 13.8 ± 2.32 | 0.131 | √ |
| Hematocrit | 39.98 ± 5.58 | 40.82 ± 6.51 | 0.282 |  |
| Erythrocyte Sedimentation Rate, mm/hr | 15.08 ± 13.82 | 14.79 ± 16.31 | 0.895 |  |
| Glucose | 145.23 ± 64.06 | 139.7 ± 68.21 | 0.509 |  |
| Total cholesterol, mg/dL | 152.01 ± 42.27 | 173.9 ± 121.41 | 0.109 |  |
| Triglyceride, mg/dL | 128.29 ± 69.88 | 146.8 ± 118.29 | 0.176 |  |
| High density lipoprotein, mg/dL | 43.16 ± 14.26 | 45.64 ± 12.27 | 0.115 |  |
| Low density lipoprotein, mg/dL | 83.67 ± 36.89 | 96.48 ± 73.17 | 0.126 |  |
| C-reactive protein, mg/dL | 0.95 ± 1.83 | 10.14 ± 143.35 | 0.590 |  |
| Ferritin, µg/L | 218.22 ± 323.02 | 189.91 ± 146.54 | 0.246 |  |
| Homocysteine, µMol/L | 16.52 ± 11.27 | 15.91 ± 7.74 | 0.567 |  |
| Rheumatoid factor, U/mL | 15.73 ± 18.83 | 14.69 ± 31.35 | 0.777 |  |
| White blood cell | 8.3 ± 3.52 | 8.17 ± 3.56 | 0.771 |  |
| Platelet count | 208.27 ± 65.19 | 233.07 ± 73.84 | 0.005* |  |
| Blood urea nitrogen, mg/dL | 17.73 ± 8.27 | 17.12 ± 15.1 | 0.725 |  |
| Creatinine mg/dL | 1.16 ± 0.91 | 1.02 ± 0.85 | 0.197 |  |
| Sodium, mMol/L | 138.9 ± 3.17 | 139.02 ± 7.71 | 0.895 |  |
| Potassium, mMol/L | 4.13 ± 0.38 | 4.13 ± 0.42 | 0.981 |  |
| Albumin, g/dL | 4.14 ± 0.45 | 4.29 ± 0.46 | 0.008* |  |
| Total bilirubin, mg/dL | 0.65 ± 0.4 | 0.59 ± 0.42 | 0.236 |  |
| Alanine aminotransferase, U/L | 19.52 ± 14.44 | 29.65 ± 117.15 | 0.431 |  |
| Aspartate aminotransferase, U/L | 27.19 ± 22.47 | 26.96 ± 42.62 | 0.962 |  |
| **Dynamic features based on vital sign** | | | | |
| Body temperature, °C | 36.97 ± 0.42 | 36.79 ± 0.43 | < 0.001* | √ |
| Heart rate, bpm | 75.49 ± 15.18 | 71.22 ± 14.36 | 0.023* | √ (mean, SD2) |
| Respiratory rate, breaths/min | 18.15 ± 3.26 | 17.45 ± 3.28 | <0.001* | √ (a2, SD1, SDSD,RMSSD) |
| Oxygen saturation, % | 96.64 ± 1.39 | 96.23 ± 3.19 | 0.271 | √ (mean, SDSD, SD, RMSSD) |
| Systolic blood pressure, mmHg | 148.24 ± 21.25 | 148.52 ± 24.18 | 0.841 |  |
| Diastolic blood pressure, mmHg | 85.16 ± 13.42 | 86.1 ± 13.92 | 0.258 | √ (SD ratio) |

**p* < 0.05, **^†^**Number of comorbidities among hypertension, dyslipidemia, or diabetes, NIHSS, National Institute of Health Stroke Scale; mRS, modified Rankin Scale; SD1/SD2, Poincaré plot standard deviation of the major/minor axis; SDSD, standard deviation of successive difference; RMSSD, root mean square of successive difference; SD, standard deviation; SD ratio, ratio between SD1 and SD2 (SD2/SD1)

**Supplementary Table 3. Characteristics of vital-sign variability**

| **Variable** | | | **Case group**  **(n = 84)** | **Control group**  **(n = 336)** | ***p*-value** |
| --- | --- | --- | --- | --- | --- |
|  | **Heart rate** | |  |  |  |
|  | | **Mean** | 75.49 ± 15.18 | 71.22 ± 14.36 | **0.023** |
|  | | Std | 5.24 ± 2.3 | 5.09 ± 2.59 | 0.341 |
|  | | SDSD | 3.47 ± 1.9 | 3.43 ± 1.86 | 0.713 |
|  | | RMSSD | 4.92 ± 2.59 | 4.79 ± 2.56 | 0.41 |
|  | | SD1 | 3.48 ± 1.83 | 3,39 ± 1.8 | 0.412 |
|  | | SD2 | 6.41 ± 2.95 | 6.25 ± 3.38 | 0.436 |
|  | | SD ratio | 2.05 ± 0.94 | 2.01 ± 0.93 | 0.497 |
|  | | Alpha1 | 0.99 ± 0.23 | 0.99 ± 0.21 | 0.722 |
|  | | Alpha2 | 0,93 ± 0.45 | 0.93 ± 0.45 | 0.298 |
|  | | Alpha ratio | 0,96 ± 0.48 | 1.0 ± 0.54 | 0.271 |
|  | | **SampEn** | 0.37 ± 0.17 | 0.35 ± 0.16 | **0.042** |
|  | **Oxygen saturation** | |  |  |  |
|  | | Mean | 96.64 ± 1.39 | 96.23 ± 3.19 | 0.271 |
|  | | **Std** | 1.25 ± 0.63 | 1.07 ± 0.57 | **<0.001** |
|  | | **SDSD** | 1.07 ± 0.66 | 0.88 ± 0.47 | **<0.001** |
|  | | **RMSSD** | 1.41 ± 0.82 | 1.16 ± 0.6 | **<0.001** |
|  | | **SD1** | 1.0 ± 0.58 | 0.82 ± 0.43 | **<0.001** |
|  | | **SD2** | 1.44 ± 0.74 | 1.33 ± 0.71 | **0.014** |
|  | | **SD ratio** | 1.56 ± 0.67 | 1.75 ± 0.84 | **<0.001** |
|  | | **Alpha1** | 0.9 ± 0.24 | 0.96 ± 0.24 | **<0.001** |
|  | | **Alpha2** | 0.81 ± 1.41 | 0.88 ± 0.44 | **0.011** |
|  | | Alpha ratio | 0.91 ± 0.56 | 0.94 ± 0.55 | 0.302 |
|  | | SampEn | 0.49 ± 0.31 | 0.48 ± 0.3 | 0.524 |
|  | **Respiratory rate** | |  |  |  |
|  | | **Mean** | 18.15 ± 3.26 | 17.45 ± 3.28 | **<0.001** |
|  | | **Std** | 3.51 ± 1.22 | 3.12 ± 1.27 | **<0.001** |
|  | | **SDSD** | 2.96 ± 0.98 | 2.57 ± 0.99 | **<0.001** |
|  | | **RMSSD** | 4.37 ± 1.45 | 3.7 ± 1.41 | **<0.001** |
|  | | **SD1** | 3.09 ± 1.03 | 2.62 ± 1.0 | **<0.001** |
|  | | **SD2** | 3.84 ± 1.45 | 3.51 ± 1.58 | **<0.001** |
|  | | **SD ratio** | 1.26 ± 0.28 | 1.36 ± 0.41 | **<0.001** |
|  | | **Alpha1** | 0.77 ± 0.16 | 0.81 ± 0.17 | **<0.001** |
|  | | **Alpha2** | 0.68 ± 0.35 | 0.77 ± 0.39 | **<0.001** |
|  | | Alpha ratio | 0.93 ± 0.55 | 0.98 ± 0.52 | 0.122 |
|  | | **SampEn** | 0.5 ± 0.14 | 0.45 ± 0.16 | **<0.001** |
|  | **Systolic blood pressure** | |  |  |  |
|  | | Mean | 148.24 ± 21.25 | 148.52 ± 24.18 | 0.841 |
|  | | Std | 6.25 ± 6.47 | 5.67 ± 5.94 | 0.103 |
|  | | **SDSD** | 1.86 ± 2.13 | 1.64 ± 1.85 | **0.049** |
|  | | **RMSSD** | 1.87 ± 2.17 | 1.65 ± 1.87 | **0.0478** |
|  | | **SD1** | 1.32± 1.53 | 1.16 ± 1.32 | **0.046** |
|  | | SD2 | 8.72 ± 9.08 | 7.86 ± 8.24 | 0.08 |
|  | | SD ratio | 7.35 ± 2.41 | 7.43 ± 2.43 | 0.65 |
|  | | Alpha1 | 1.84 ± 0.58 | 1.82 ± 0.54 | 0.483 |
|  | | Alpha2 | 1.6 ± 2.59 | 1.6 ± 2.38 | 0.961 |
|  | | Alpha ratio | 0.86 ± 1.68 | 1.04 ± 8.98 | 0.772 |
|  | | SampEn | 0.03 ± 0.02 | 0.03 ± 0,02 | 0.635 |
|  | **Diastolic blood pressure** | |  |  |  |
|  | | Mean | 85.16 ± 13.42 | 86.1 ± 13.92 | 0.258 |
|  | | Std | 3.94 ± 3.89 | 3.58 ± 3.85 | 0.116 |
|  | | **SDSD** | 1.18 ± 1.33 | 1.03 ± 1.19 | **0.041** |
|  | | **RMSSD** | 1.18 ± 1.34 | 1.03 ± 1.2 | **0.041** |
|  | | **SD1** | 0.83 ± 0.95 | 0.73 ± 0.85 | **0.04** |
|  | | SD2 | 5.5 ± 5.34 | 4.97 ± 5.35 | 0.102 |
|  | | SD ratio | 7.47 ± 2.42 | 7.48 ± 2.44 | 0.913 |
|  | | Alpha1 | 1.81 ± 0.55 | 1.82 ± 0.56 | 0.965 |
|  | | Alpha2 | 1.63 ± 2.4 | 1.58 ± 2.52 | 0.778 |
|  | | Alpha ratio | 0.94 ± 2.15 | 0.76 ± 7.12 | 0.711 |
|  | | SampEn | 0.03 ± 0.02 | 0.03 ± 0.02 | 0.807 |
|  | **Heart rate variability (calculated by respiratory rate interval from electrocardiogram)** | | | | |
|  | | SNDD | 317 ± 1080 | 324.66 ± 1830 | 0.947 |
|  | | RMSSD | 403 ± 1436 | 396.31 ± 2243 | 0.962 |
|  | | SDSD | 409.83 ± 1449 | 408.61 ± 2459 | 0.994 |
|  | | NN50 | 251.29 ± 437.1 | 182.22 ± 355 | **0.003** |
|  | | pNN50 | 13.6 ± 21.53 | 9.59 ± 17.51 | **<0.001** |
|  | | NN20 | 80.85 ± 187.01 | 51.89 ± 124.67 | **<0.001** |
|  | | pNN20 | 5.18 ± 12.28 | 3.25 ± 8.08 | **<0.001** |
|  | | VLF | 69.15 ± 162 | 90.64 ± 452.97 | 0.447 |
|  | | LF | 173.73 ± 225.6 | 243.36 ± 1256 | 0.373 |
|  | | HF | 347.71 ± 358 | 529.13 ± 2790 | 0.296 |
|  | | LF/HF | 0.49 ± 0.25 | 0.47 ± 0.17 | **0.0421** |
|  | | SD1 | 289.78 ± 1025 | 288.37 ± 1731 | 0.99 |
|  | | SD2 | 325.34 ± 1122 | 313.48 ± 1652 | 0.909 |
|  | | SD ratio | 0.71 ± 0.34 | 0.63 ± 0.33 | **<0.001** |
|  | | Alpha1 | 0.96 ± 0.43 | 1.02 ± 0.34 | **0.005** |
|  | | Alpha2 | 0.97 ± 0.47 | 1.01 ± 0.31 | **0.082** |
|  | | Alpha ratio | 1.01 ± 0.47 | 1.05 ± 0.78 | 0.393 |
|  | | SampEn | 0.84 ± 0.63 | 0.87 ± 0.62 | 0.497 |

SD1/SD2, Poincaré plot standard deviation of the major/minor axis; SDSD, standard deviation of successive difference; RMSSD, root mean square of successive difference

**Supplementary Table 4. Comparison of a model using only EMR data and a model using EMR and vital-sign data**

| **Data** | **AUROC**  **(95% CI)** | **AUPRC**  **(95% CI)** | **Accuracy**  **(95% CI**) | **Precision**  **(95% CI)** | **Recall**  **(95% CI)** | **F1 score**  **(95% CI)** | **Sensitivity**  **(95% CI)** | **Specificity**  **(95% CI)** |
| --- | --- | --- | --- | --- | --- | --- | --- | --- |
| Features at admission | 0.70  (0.68–0.72) | 0.40  (0.37–0.43) | 0.68  (0.65–0.71) | 0.372  (0.35–0.394) | 0.68  (0.63–0.72) | 0.46  (0.44–0.48) | 0.68  (0.63–0.72) | 0.68  (0.64–0.72) |
| **Features at admission + Vital sign** | **0.80**  **(0.78–0.81)** | **0.55**  **(0.52–0.58)** | **0.73**  **(0.71–0.75)** | **0.42**  **(0.4–0.44)** | **0.75**  **(0.72–0.79)** | **0.53**  **(0.51–0.55)** | **0.75**  **(0.72–0.79)** | **0.72**  **(0.69–0.75)** |

AUROC, area under the receiver operating characteristic curve; AUPRC, area under the precision-recall curve; CI, confidence interval; EMR, electronic medical record

**Supplementary Table 5. Comparison of performance over the different sources of features (AUC)**

|  | **Fixed features at admission** | **Vital sign-based dynamic features** | **Fixed + dynamic features** | **Fixed vs. dynamic^†^** | **Fixed vs. both^†^** | **Dynamic vs. both^†^** |
| --- | --- | --- | --- | --- | --- | --- |
| 1 | 0.6 | 0.67 | 0.75 | 0.49 | 0.03 | 0.23 |
| 2 | 0.7 | 0.7 | 0.77 | 0.97 | 0.36 | 0.46 |
| 3 | 0.67 | 0.71 | 0.77 | 0.73 | 0.01 | 0.46 |
| 4 | 0.75 | 0.58 | 0.81 | 0.08 | 0.27 | <0.01 |
| 5 | 0.58 | 0.73 | 0.71 | 0.23 | 0.18 | 0.79 |
| 6 | 0.62 | 0.62 | 0.71 | 0.99 | 0.19 | 0.20 |
| 7 | 0.77 | 0.76 | 0.85 | 0.81 | 0.19 | 0.01 |
| 8 | 0.77 | 0.67 | 0.86 | 0.32 | 0.13 | <0.01 |
| 9 | 0.7 | 0.8 | 0.86 | 0.27 | <0.01 | 0.24 |
| 10 | 0.76 | 0.75 | 0.84 | 0.85 | 0.19 | 0.05 |
| 11 | 0.64 | 0.72 | 0.7 | 0.48 | 0.21 | 0.84 |
| 12 | 0.77 | 0.74 | 0.84 | 0.77 | 0.23 | 0.12 |
| 13 | 0.63 | 0.74 | 0.75 | 0.27 | 0.05 | 0.90 |
| 14 | 0.74 | 0.67 | 0.84 | 0.49 | 0.13 | 0.01 |
| 15 | 0.7 | 0.7 | 0.73 | 0.96 | 0.54 | 0.80 |
| 16 | 0.65 | 0.72 | 0.88 | 0.46 | <0.01 | 0.01 |
| 17 | 0.74 | 0.68 | 0.82 | 0.49 | 0.16 | 0.01 |
| 18 | 0.68 | 0.66 | 0.77 | 0.85 | 0.04 | 0.19 |
| 19 | 0.57 | 0.8 | 0.83 | 0.01 | <0.01 | 0.64 |
| 20 | 0.63 | 0.77 | 0.8 | 0.20 | 0.01 | 0.74 |
| 21 | 0.66 | 0.6 | 0.76 | 0.55 | 0.06 | 0.04 |
| 22 | 0.57 | 0.68 | 0.69 | 0.24 | 0.03 | 0.96 |
| 23 | 0.59 | 0.64 | 0.72 | 0.66 | 0.05 | 0.22 |
| 24 | 0.78 | 0.67 | 0.79 | 0.37 | 0.90 | 0.26 |
| 25 | 0.73 | 0.75 | 0.85 | 0.75 | 0.06 | 0.05 |
| 26 | 0.63 | 0.64 | 0.64 | 0.96 | 0.84 | 0.99 |
| 27 | 0.8 | 0.64 | 0.77 | 0.11 | 0.46 | 0.14 |
| 28 | 0.74 | 0.82 | 0.85 | 0.20 | 0.01 | 0.57 |
| 29 | 0.65 | 0.71 | 0.71 | 0.63 | 0.17 | 0.92 |
| 30 | 0.62 | 0.65 | 0.73 | 0.76 | 0.05 | 0.18 |
| 31 | 0.82 | 0.71 | 0.84 | 0.24 | 0.74 | <0.01 |
| 32 | 0.69 | 0.77 | 0.77 | 0.46 | 0.14 | 0.97 |
| 33 | 0.73 | 0.83 | 0.92 | 0.27 | <0.01 | 0.08 |
| 34 | 0.66 | 0.71 | 0.81 | 0.63 | 0.05 | 0.10 |
| 35 | 0.61 | 0.71 | 0.76 | 0.30 | 0.02 | 0.28 |
| 36 | 0.79 | 0.68 | 0.79 | 0.29 | 0.94 | 0.09 |
| 37 | 0.79 | 0.77 | 0.85 | 0.79 | 0.22 | 0.12 |
| 38 | 0.8 | 0.72 | 0.88 | 0.45 | 0.08 | 0.01 |
| 39 | 0.72 | 0.7 | 0.77 | 0.89 | 0.35 | 0.35 |
| 40 | 0.61 | 0.73 | 0.74 | 0.21 | 0.01 | 0.93 |
| 41 | 0.82 | 0.65 | 0.86 | 0.13 | 0.17 | 0.03 |
| 42 | 0.84 | 0.75 | 0.88 | 0.25 | 0.33 | 0.03 |
| 43 | 0.67 | 0.65 | 0.72 | 0.89 | 0.40 | 0.38 |
| 44 | 0.67 | 0.74 | 0.82 | 0.54 | 0.05 | 0.31 |
| 45 | 0.72 | 0.68 | 0.85 | 0.67 | 0.08 | <0.01 |
| 46 | 0.75 | 0.73 | 0.86 | 0.82 | 0.09 | 0.11 |
| 47 | 0.69 | 0.74 | 0.84 | 0.56 | <0.01 | 0.13 |
| 48 | 0.71 | 0.73 | 0.82 | 0.79 | 0.03 | 0.18 |
| 49 | 0.72 | 0.72 | 0.8 | 0.98 | 0.17 | 0.35 |
| 50 | 0.73 | 0.75 | 0.86 | 0.79 | 0.07 | <0.01 |
| **Average** | **0.70** | **0.71** | **0.80** |  |  |  |
| **Fixed vs. dynamic^‡^** |  |  |  | 0.45 |  |  |
| **Fixed vs. both^‡^** |  |  |  |  | <0.01 |  |
| **Dynamic vs. both^‡^** |  |  |  |  |  | <0.01 |

(AUC: Area under the curve, ^†^Delong test, ^‡^Student’s t-test)

**Supplementary Table 6. Model prediction probability and total number of alarms**

| **Onset before** | | **0–2 h** | **2–4 h** | **4–6 h** | **6–8 h** | **8–10 h** | **10–12 h** | **12–14 h** | **14–16 h** |
| --- | --- | --- | --- | --- | --- | --- | --- | --- | --- |
| **Delirium** | Total patients | 850 | 787 | 716 | 660 | 590 | 571 | 566 | 531 |
|  | Total alarm | 580 | 535 | 485 | 443 | 339 | 332 | 321 | 333 |
|  | **Mean probability** | **0.41** | **0.39** | **0.361** | **0.375** | **0.339** | **0.351** | **0.329** | **0.35** |
|  | **Alarm per patient** | **0.682** | **0.68** | **0.677** | **0.671** | **0.575** | **0.581** | **0.567** | **0.627** |
| **Non-delirium** | Total patients | 3,940 | 3,301 | 3,102 | 2,901 | 2,691 | 2,592 | 2,532 | 2,397 |
|  | Total alarm | 1,232 | 1,126 | 1,138 | 1,079 | 946 | 958 | 954 | 939 |
|  | **Mean probability** | **0.194** | **0.215** | **0.218** | **0.228** | **0.224** | **0.233** | **0.235** | **0.245** |
|  | **Alarm per patient** | **0.313** | **0.341** | **0.367** | **0.372** | **0.352** | **0.37** | **0.377** | **0.392** |
